# Associations of serum 25-hydroxyvitamin D with metabolic syndrome and its components in elderly men and women: the Korean Urban Rural Elderly cohort study

**DOI:** 10.1186/s12877-019-1118-y

**Published:** 2019-04-11

**Authors:** Su Jin Lee, Eun Young Lee, Jung Hyun Lee, Jong Eun Kim, Kwang Jun Kim, Yumie Rhee, Hyeon Chang Kim, Yoosik Youm, Chang Oh Kim

**Affiliations:** 1 Department of Internal Medicine, Severance Hospital, Endocrine Research Institute, Yonsei University College of Medicine, Seoul, South Korea; 2 Department of Preventive Medicine, Yonsei University College of Medicine, 50-1 Yonsei-ro, Seodaemun-gu, Seoul, South Korea; 3 Division of Endocrinology and Metabolism, Department of Internal Medicine, Seoul St. Mary’s Hospital, College of Medicine, The Catholic University of Korea, Seoul, South Korea; 4 Graduate School of Public Health Yonsei University, Seoul, South Korea; 5 Department of Sociology, Yonsei University, 50 Yonsei-ro, Seodaemun-gu, Seoul, South Korea; 6 Division of Geriatrics, Department of Internal Medicine, Yonsei University College of Medicine, 50-1 Yonsei-ro, Seodaemun-gu, Seoul, 03772 Republic of Korea

**Keywords:** Vitamin D, Metabolic syndrome, Elderly, High waist circumference, Hypertriglyceridemia, Low high-density lipoprotein

## Abstract

**Background:**

Many studies have investigated the association between vitamin D and metabolic syndrome (MetS). However, few studies have investigated the association stratified by sex in the elderly. Therefore, we aimed to evaluate the association between vitamin D, MetS, and its components in Korean elderly men and women.

**Methods:**

A total of 987 men and 1949 women aged ≥65 years were recruited through Korean Urban Rural Elderly cohort study. Serum 25-hydroxyvitamin D (25(OH)D) levels were categorized into 4 quartiles and all data were analyzed separately by sex. MetS was defined by the revised criteria of the National Cholesterol Education Program Adult Treatment Panel III.

**Results:**

The participants in the lowest quartile of serum 25(OH)D showed a significant increase in the prevalence of high waist circumference, elevated triglyceride level, and low high-density lipoprotein cholesterol level, as well as MetS itself, in both men and women in a univariate analysis. After adjusting for potential confounders including age, smoking status, drinking status, exercise status, region of residence, seasonality, and parathyroid hormone level, the lowest 25(OH)D quartile group was associated with a higher risk of MetS (odds ratio [OR] 2.25, 95% confidence interval [CI] 1.48–3.43 in men and OR 1.65, 95% CI 1.27–2.16 in women) compared to the highest 25(OH)D quartile group as the reference group. However, no significant association was found between serum 25(OH)D levels and the prevalence of MetS components including hyperglycemia or hypertension in both men and women.

**Conclusions:**

Low 25(OH)D levels were associated with increased odds of MetS; in particular, they were associated with MetS components of high waist circumference, hypertriglyceridemia, and low high-density lipoprotein-cholesterol, after adjusting for age, smoking, alcohol, exercise, region of residency, and seasonality, in men and women over 65 years old.

**Electronic supplementary material:**

The online version of this article (10.1186/s12877-019-1118-y) contains supplementary material, which is available to authorized users.

## Background

Vitamin D deficiency is a frequent public health issue in the elderly worldwide. In Korea, vitamin D deficiency was found in 47.3% of men and 64.5% females in 2008 [1]. More than 50% of women and 30% men had vitamin D levels below 20 ng/mL among individuals in their 60s and older populations in 2008 [1]. Vitamin D plays an important role in various physiological functions including maintaining calcium homeostasis and bone health. As vitamin D receptors are distributed in most of tissues like the muscle, pancreas, brain, prostate, and breast, among others, vitamin D is also involved in various extra-skeletal functions [2–4]. In the elderly, vitamin D deficiency is not only considered the risk factor of osteoporosis and fractures but also is associated with beta cell dysfunction and increased insulin resistance leading to cardiometabolic diseases such as diabetes and metabolic syndrome (MetS) [3–6].

MetS is a cluster of factors that have insulin resistant characteristics such as high waist circumference, high blood pressure, high blood sugar, and hyperlipidemia. MetS is one of the major public health concerns with increasing prevalence. The prevalence of MetS was 31.3% in 2007 in Korea. Notably, the prevalence of MetS was the highest in individuals in their 60s with as high as > 70% in women and approximately 50% in men [7]. Because vitamin D deficiency causes insulin resistance which is linked to MetS, the associations between vitamin D deficiency and MetS have been studied [8–10]. Many studies have revealed that vitamin D deficiency increases the prevalence of MetS in various populations. However, the results about the associations between the vitamin D level and each component of MetS in different populations are inconsistent [9, 11]. Moreover, there were few studies focusing on the association of vitamin D level and MetS by sex-stratification over 65 years old. Thus, we aimed to elucidate the association of MetS and its components on the basis of vitamin D levels in elderly Korean men and women, separately.

## Methods

### Study population and data collection

We studied 2936 participants including 987 men and 1949 women aged ≥65 years from the Korean Urban Rural Elderly cohort study [12]. Serum 25-hydroxyvitamin D (25(OH)D) levels were categorized into 4 quartiles for both men and women. The revised criteria of the National Cholesterol Education Program Adult Treatment Panel III (NCEP-ATP III) were used to define MetS [13, 14]. MetS was diagnosed if ≥3 of the following criteria were met: (i) high waist circumference, defined as a waist circumference ≥ 90 cm for men or ≥ 80 cm for women; (ii) hypertriglyceridemia, defined as serum triglyceride level ≥ 150 mg/dL; (iii) low high-density lipoprotein (HDL) cholesterol, defined as a serum HDL cholesterol level < 40 mg/dL for men or < 50 mg/dL for women; (iv) hypertension, defined as a systolic blood pressure ≥ 130 mmHg, diastolic blood pressure ≥ 85 mmHg, or the use of an antihypertensive drug; (v) hyperglycemia, defined as fasting glucose ≥100 mg/dL or the use of an antidiabetic drug. The study protocol was approved by the Ethics Committee of Severance Hospital (Seoul, Korea) (IRB No. 4–2012-0172).

### Biochemical measurements and questionnaires

All blood samples were collected after an overnight fast (> 8-h fasting) and analyzed within 24 h. Plasma glucose and creatinine levels were measured by performing a colorimetry immunoassay, and serum triglyceride, HDL cholesterol, and total cholesterol levels were also measured using an enzymatic method using an ADVIA 1800 Auto Analyzer (Siemens Medical Solutions, Malvern, PA, USA). Serum insulin level was measured by performing a chemiluminescence immunoassay using an ADVIA Centaur XP (Siemens Medical Solutions, Malvern, PA, USA). Serum 25(OH)D level was measured by using a chemiluminescence immunoassay (Liaison; DiaSorin, Dietzenbach, Germany). Intra- and interassay coefficients of variation ranged from 2.9 to 5.5% and from 6.5 to 12.9%, respectively. Estimated glomerular filtration rate (eGFR) was calculated using the CKD-EPI [15]. Parathyroid hormone (PTH) level was measured by using an electrochemiluminescence immunoassay (E-170 modular, Roche, USA). Total coefficients of variation ranged from 2.8 to 3.4%. Homeostatic model assessment-insulin resistance (HOMA-IR) was calculated by using the following equation: [fasting plasma insulin (μIU/mL) × fasting plasma glucose (mg/dL)/405] [16]. At the first visit, self-reported questionnaires on patient medical histories, smoking status (current smoker or not), alcohol drinking status (current drinker or not), and exercise (currently involved in exercise or not) were administered.

### Statistical analysis

All analyses were stratified by sex. Data were presented as mean ± standard deviation or median [25th quartile – 75th quartile], or numbers (n) and percentages (%). Analysis of variance (ANOVA) and Kruskal-Wallis tests were performed to compare groups on the basis of quartile of serum 25(OH)D level. Categorical variables were analyzed by using the χ^2^ or Mantel-Haenszel χ^2^ test. Trend test was performed for MetS and its components on the basis of the quartile of serum 25(OH)D. Univariate regression analysis was used to evaluate the associations of serum 25(OH)D levels with MetS and its components including high waist circumference, hypertension, hyperglycemia, hypertriglyceridemia, and low HDL cholesterol, respectively. Multivariate logistic regression analyses were conducted to estimate the associations between vitamin D status and MetS, as well as between vitamin D status and each component of MetS. Model 1 was adjusted by age. Model 2 was adjusted by age, exercise status, and smoking status. Model 3 was adjusted by age, exercise status, smoking status, region of residence, and seasonality. Model 4 was adjusted by PTH level in addition to the covariates adjusted for in model 3. All statistical analyses were performed using SAS software, version 9.4 (SAS Institute Inc., Cary, NC, USA). A *p*-value of < 0.05 was considered to be statistically significant.

## Results

### Baseline characteristics

The mean age of 2936 people in total was 71.9 ± 4.6 years (72.8 ± 4.6 years in men and 71.5 ± 4.6 years in women, respectively). Overall, the mean 25(OH)D level was 18.0 ± 7.8 ng/mL. The level of serum 25(OH)D was higher in men than those in women (19.7 ± 7.5 ng/mL in men and 17.1 ± 7.9 ng/mL in women, *p* <  0.0001). To evaluate the relationship between vitamin D levels and MetS, the study population was stratified according to the quartiles of serum 25(OH)D level for each sex. Clinical and biochemical characteristics of the participants divided into 4 groups stratified by sex are shown in Tables 1 and 2, respectively. For both sexes, waist circumference, PTH level, insulin level, and HOMA-IR, total cholesterol level, and triglyceride level were higher in the lowest 25(OH)D quartile, compared with the highest quartile of 25(OH)D. There were no differences in age, body mass index, systolic and diastolic blood pressure, eGFR, and HDL cholesterol level among the 4 groups. The life style parameters such as smoking and drinking did not significantly differ among the 4 groups for both sexes. The number of MetS components was highest in the lowest 25(OH)D quartile and lowest in the highest 25(OH)D quartile in both sexes.

**Table 1:** Baseline characteristics of men according to serum 25(OH)D level

Quartile (n) (ng/mL)	Quartile 1 (*n* = 245) (4.20–14.19)	Quartile 2 (*n* = 245) (14.20–18.99)	Quartile 3 (*n* = 249) (19.00–24.19)	Quartile 4 (*n* = 248) (24.20–51.90)	*P* value
25(OH)D, ng/mL	10.90 ± 2.28	16.53 ± 1.39	21.41 ± 1.50	29.67 ± 5.34	< 0.001
Age, yr	73.01 ± 4.87	72.38 ± 4.14	72.59 ± 4.47	73.27 ± 4.87	0.132
BMI, kg/m^2^	23.99 ± 2.63	24.19 ± 2.61	23.65 ± 2.67	23.60 ± 2.90†	0.053
Waist, cm	87.49 ± 8.13	86.68 ± 8.44	86.43 ± 8.78	83.48 ± 9.33*†‡	< 0.001
SBP, mmHg	125.89 ± 14.50	128.51 ± 14.24	129.70 ± 14.41	128.86 ± 13.88	0.370
DBP, mmHg	74.10 ± 8.91	73.68 ± 9.28	75.23 ± 8.35	73.02 ± 7.90	0.420
Current smoking	42 (17.1)	53 (21.6)	36 (14.5)	53 (21.4)	0.117
Current drinking	205 (66.6)	194 (64.2)	138 (58)	72 (51.8)	0.011
Exercise	151 (61.4)	164 (66.9)	164 (65.9)	132 (53.2)	0.007
eGFR	77.5 ± 15.11	79.8 ± 14.03	81.06 ± 13.19*	80.13 ± 13.44	0.036
PTH	39.68 ± 14.13	38.07 ± 17.25	35.80 ± 12.07	33.83 ± 11.62*†	< 0.001
Glucose, mg/dL	101.59 ± 23.61	100.47 ± 20.17	99.91 ± 19.90	97.50 ± 19.93	0.171
Insulin, IU/L	7.53 ± 8.31	7.57 ± 12.22	6.12 ± 4.26	5.18 ± 3.26*†	0.001
HOMA-IR	1.93 ± 2.24	1.86 ± 2.42	1.55 ± 1.24	1.29 ± 0.99*†	< 0.001
Total cholesterol, mg/dL	174.51 ± 32.91	172.92 ± 33.72	173.93 ± 33.09	163.70 ± 29.95*†‡	< 0.001
Triglyceride, mg/dL	138.69 ± 78.55	128.43 ± 71.30	125.45 ± 61.22	110.31 ± 46.04*†	< 0.001
HDL cholesterol, mg/dL	45.99 ± 12.13	47.89 ± 12.56	46.98 ± 10.45	48.15 ± 11.26	0.157
Number of MetS components	2.22 ± 1.30	2.16 ± 1.23	2.02 ± 1.25	1.73 ± 1.18*†	< 0.001

Data were shown as mean ± SD or number (%). 25(OH)D, vitamin D3, *BMI* body mass index, *SBP* systolic blood pressure, *DBP* diastolic blood pressure, *eGFR* estimated glomerular filtration rate, *HOMA-IR* homeostatic model assessment - insulin resistance, *HDL* high density lipoprotein, *MetS* metabolic syndrome. * vs. Q1, † vs. Q2, ‡ vs. Q3

**Table 2:** Baseline characteristics of women according to serum 25(OH)D level

Quartile (n) (ng/mL)	Quartile 1 (*n* = 487) (4.10–11.19)	Quartile 2 (*n* = 491) (11.20–15.59)	Quartile 3 (*n* = 484) (15.60–21.59)	Quartile 4 (*n* = 487) (21.60–54.90)	*P* value
25(OH)D, ng/mL	8.72 ± 1.67	13.28 ± 1.28	18.31 ± 1.70	28.15 ± 5.56	< 0.001
Age, yr	72.16 ± 4.68	71.07 ± 4.71	71.27 ± 4.35	71.45 ± 4.54*†‡	0.001
BMI, kg/m^2^	24.69 ± 2.92	25.07 ± 3.31	24.74 ± 3.11	23.58 ± 2.85*†‡	< 0.001
Waist, cm	83.49 ± 8.71	83.63 ± 9.13	83.14 ± 8.52	81.07 ± 8.54*†‡	< 0.001
SBP, mmHg	129.69 ± 15.75	128.51 ± 16.42	125.83 ± 15.46	124.69 ± 15.21*	0.026
DBP, mmHg	74.15 ± 7.96	73.32 ± 8.65	72.55 ± 8.75	72.96 ± 9.28	0.451
Current smoking	7 (1.4)	12 (2.4)	5 (1)	5 (1)	0.216
Current drinking	99 (23.2)	91 (20.6)	145 (28.4)	137 (24)	0.043
Exercise	293 (60.2)	308 (62.7)	294 (60.7)	294 (60.4)	0.855
eGFR	81.04 ± 15.49	82.51 ± 13.94	82.74 ± 13.23	83.68 ± 11.50*	0.024
PTH	46.36 ± 19.35	41.12 ± 14.40	37.70 ± 14.11	36.28 ± 13.80*†	< 0.001
Glucose, mg/dL	96.11 ± 16.34	99.40 ± 20.33	98.17 ± 19.57	95.76 ± 15.56†	0.004
Insulin, IU/L	7.63 ± 4.79	8.09 ± 5.69	7.60 ± 5.07	6.85 ± 7.55†	0.011
HOMA-IR	1.85 ± 1.35	2.07 ± 1.67	1.89 ± 1.47	1.68 ± 2.23†	0.005
Total cholesterol, mg/dL	190.70 ± 38.79	185.70 ± 32.22	187.60 ± 36.56	180.07 ±34.45*‡	< 0.001
Triglyceride, mg/dL	143.47 ± 72.92	127.91 ± 61.37	128.05 ± 57.94	115.83 ± 51.41*†‡	< 0.001
HDL cholesterol, mg/dL	49.85 ± 12.58	51.63 ± 12.61	52.29 ± 12.13	52.30 ± 12.12*	0.005
Number of MetS components	2.65 ± 1.29	2.52 ± 1.30	2.50 ± 1.32	2.26 ± 1.31*†‡	< 0.001

Data were shown as mean ± SD or number (%). 25(OH)D, vitamin D3, *BMI* body mass index, *SBP* systolic blood pressure, *DBP* diastolic blood pressure, *eGFR* estimated glomerular filtration rate, *HOMA-IR* homeostatic model assessment - insulin resistance, *HDL* high density lipoprotein, *MetS* metabolic syndrome. * vs. Q1, † vs. Q2, ‡ vs. Q3

### Association of serum 25(OH)D levels with MetS and its components

**Fig. 1:**
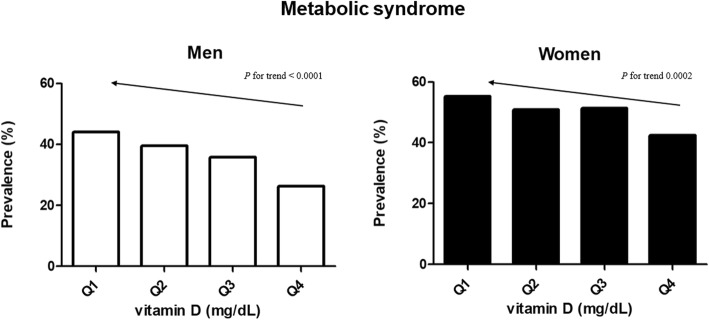
The prevalence of metabolic syndrome and its components, on the basis of serum 25(OH)D level

Among all participants, 45.4% of participants had MetS. The prevalence of MetS was higher in women (50.0%) than in men (36.4%). The prevalence of MetS according to quartile of vitamin D was presented in Fig. 1. As the 25(OH)D level decreased, the prevalence of MetS increased in both sexes (*p* for trend < 0.001, both sexes) (Fig. 1). The prevalence of high waist circumference, hypertriglyceridemia, and low HDL cholesterol significantly increased with decreasing vitamin D level; however, the prevalence of hyperglycemia showed this trend only in men (*p* for trend = 0.011) (Table 3). Hypertension did not show a significant change with decreasing level of vitamin D in both sexes. On univariate analysis (Table 3), the odds of MetS significantly increased in the lowest quartile of 25(OH)D level (OR 2.22 [95% CI, 1.52–2.34] in men and 1.98 [95% CI, 1.60–2.44] in women). To control potential confounding factors that affect the outcome in addition to age; smoking, alcohol, and exercise statuses; seasonality, which could affect serum 25(OH)D level depending on the time when the examination was performed; and region of residence in which the participants live (e.g., rural or urban), were further adjusted in model 3 (Tables 4 and 5). The odds of MetS, high waist circumference, hypertriglyceridemia, and low HDL in the lower quartile groups were higher compared to those in the highest quartile group of 25(OH)D level in both men and women. With further adjustment for PTH in model 4, the pattern was similar; however, the association of high waist circumference in lowest quartile of serum 25(OH)D level disappeared in women (OR 1.28 [95% CI, 0.97–1.68]), however, was persisted in men (OR 1.81 [95% CI, 1.19–2.77.

**Table 3:** Association of serum 25(OH)D levels with metabolic syndrome and its components in men and women

25(OH)D	Metabolic syndrome	Abdominal obesity	Hypertension	Hyperglycemia	Hypertriglyceridemia	low HDL
Men			Univariate OR			
Quartile 1	2.22 (1.52–3.24)	2.03 (1.39–2.99)	0.96 (0.65–1.43)	1.48 (1.03–2.13)	2.13 (1.40–3.24)	1.77 (1.18–2.63)
Quartile 2	1.85 (1.26–2.70)	1.93 (1.32–2.84)	1.05 (0.70–1.56)	1.72 (1.20–2.47)	1.78 (1.16–2.74)	1.35 (0.90–2.03)
Quartile 3	1.57 (1.07–2.30)	1.73 (1.17–2.54)	1.05 (0.71–1.56)	1.26 (0.88–1.82)	1.78 (1.16–2.73)	1.07 (0.70–1.62)
Quartile 4	1.00 (Reference)	1.00 (Reference)	1.00 (Reference)	1.00 (Reference)	1.00 (Reference)	1.00 (Reference)
P for trend	< 0.001	< 0.001	0.860	0.011	0.001	0.002
Women Univariate OR						
Quartile 1	1.98 (1.60–2.44)	1.42 (1.09–1.84)	1.30 (0.98–1.72)	1.11 (0.85–1.45)	2.13 (1.60–2.83)	1.37 (1.06–1.76)
Quartile 2	1.40 (1.09–1.81)	1.35 (1.04–1.74)	1.20(0.91–1.58)	1.30 (1.00–1.69)	1.43 (1.07–1.92)	1.12 (0.87–1.44)
Quartile 3	1.43 (1.11–1.85)	1.43 (1.10–1.85)	1.21 (0.92–1.60)	1.17 (0.90–1.53)	1.43 (1.06–1.92)	1.05 (0.81–1.35)
Quartile 4	1.00 (Reference)	1.00 (Reference)	1.00 (Reference)	1.00 (Reference)	1.00 (Reference)	1.00 (Reference)
P for trend	< 0.001	0.017	0.082	0.343	0.001	0.013

25(OH)D: 25-OH-vitamin D, *OR* odds ratio, *CI* confidence interval, *HDL* high-density lipoprotein

**Table 4:** Multivariable-adjusted odds ratio of metabolic syndrome and its components according to serum 25(OH)D levels in men

Men	Vitamin D level						
	1st quartile		2nd quartile		3rd quartile		4th quartile
	OR	CI	OR	CI	OR	CI	
Metabolic syndrome							
Model 1	2.22	(1.52 – 3.24)	1.84	(1.26 – 2.70)	1.56	(1.06 – 2.30)	1 (Reference)
Model 2	2.27	(1.55 – 3.33)	1.87	(1.27 – 2.75)	1.55	(1.05 – 2.28)	
Model 3	2.03	(1.34 – 3.07)	1.70	(1.14 – 2.55)	1.45	(0.98 – 2.16)	
Model 4	2.25	(1.48 – 3.43)	1.83	(1.22 – 2.74)	1.51	(1.02 – 2.24)	
Abdominal obesity							
Model 1	2.03	(1.38 – 2.98)	1.92	(1.31 – 2.83)	1.72	(1.17 – 2.53)	1
Model 2	2.10	(1.43 –3.10)	1.99	(1.35 – 2.93)	1.73	(1.17 – 2.56)	
Model 3	1.81	(1.19 – 2.75)	1.79	(1.19 – 2.68)	1.64	(1.10 – 2.44)	
Model 4	1.81	(1.19 – 2.77)	1.79	(1.19 – 2.69)	1.64	(1.10 – 2.45)	
Hypertension							
Model 1	0.97	(0.65 – 1.44)	1.07	(0.72 – 1.60)	1.07	(0.72 – 1.59)	1
Model 2	1.03	(0.69 – 1.55)	1.10	(0.73 – 1.65)	1.05	(0.70 – 1.57)	
Model 3	1.05	(0.68 – 1.62)	1.10	(0.72 – 1.69)	1.04	(0.69 – 1.57)	
Model 4	1.07	(0.69 – 1.67)	1.12	(0.73 – 1.72)	1.05	(0.69 – 1.59)	
Hyperglycemia							
Model 1	1.48	(1.03 – 2.13)	1.72	(1.20 – 2.47)	1.26	(0.88 – 1.82)	1
Model 2	1.49	(1.03 – 2.14)	1.69	(1.17 – 2.44)	1.22	(0.85 – 1.76)	
Model 3	1.18	(0.79 – 1.75)	1.42	(0.97 – 2.09)	1.10	(0.75 – 1.60)	
Model 4	1.34	(0.90 – 2.01)	1.57	(1.06 – 2.31)	1.15	(0.79 – 1.68)	
Hypertriglycemia							
Model 1	2.11	(1.38 – 3.23)	1.74	(1.13 – 2.67)	1.74	(1.14 – 2.68)	1
Model 2	2.27	(1.47 – 3.48)	1.79	(1.16 – 2.76)	1.83	(1.19 – 2.82)	
Model 3	2.41	(1.51 – 3.86)	1.87	(1.19 – 2.96)	1.89	(1.21 – 2.94)	
Model 4	2.77	(1.72 – 4.48)	2.05	(1.29 – 3.25)	1.99	(1.28 – 3.11)	
Low HDL							
Model 1	1.76	(1.18 – 2.63)	1.34	(0.89 – 2.02)	1.06	(0.70 – 1.62)	1
Model 2	1.61	(1.07 – 2.41)	1.30	(0.86 – 1.97)	1.04	(0.68 – 1.59)	
Model 3	1.55	(1.00 – 2.41)	1.28	(0.83 – 1.98)	1.04	(0.67 – 1.61)	
Model 4	1.70	(1.09 – 2.67)	1.36	(0.88 – 2.12)	1.07	(0.69 – 1.66)	

Model 1: age adjusted, Model 2: Model 1 + smoking, alcohol, exercise adjusted, Model 3: Model 2 + region, seasonality adjusted, Model 4: Model 3 + parathyroid hormone adjusted, *OR* odds ratio, *CI* confidence interval, *HDL* high density lipoprotein

**Table 5:** Multivariable-adjusted odds ratio of metabolic syndrome and its components according to serum 25(OH)D levels in women

Women	Vitamin D level						
	1st quartile		2nd quartile		3rd quartile		4th quartile
	OR	CI	OR	CI	OR	CI	
Metabolic syndrome							
Model 1	1.63	(1.27 – 2.11)	1.43	(1.11 – 1.84)	1.45	(1.12 – 1.86)	1 (reference)
Model 2	1.35	(1.27 – 2.12)	1.45	(1.12 – 1.86)	1.45	(1.12 – 1.87)	
Model 3	1.61	(1.24 – 2.09)	1.42	(1.10 – 1.84)	1.43	(1.11 – 1.84)	
Model 4	1.65	(1.27 – 2.16)	1.44	(1.12 – 1.87)	1.43	(1.11 – 1.85)	
Abdominal obesity							
Model 1	1.39	(1.07 – 1.81)	1.36	(1.05 – 1.76)	1.44	(1.11 – 1.86)	1
Model 2	1.39	(1.08 – 1.81)	1.36	(1.05 – 1.77)	1.44	(1.11 – 1.87)	
Model 3	1.33	(1.02 – 1.74)	1.33	(1.03 – 1.73)	1.42	(1.09 – 1.84)	
Model 4	1.28	(0.97 – 1.68)	1.30	(1.00 – 1.70)	1.41	(1.08 – 1.83)	
Hypertension							
Model 1	1.24	(0.94 – 1.65)	1.24	(0.94 – 1.64)	1.23	(0.93 – 1.63)	1
Model 2	1.24	(0.94 – 1.65)	1.24	(0.94 – 1.64)	1.23	(0.93 – 1.63)	
Model 3	1.30	(0.97 – 1.73)	1.27	(0.96 – 1.68)	1.25	(0.94 – 1.66)	
Model 4	1.24	(0.92 – 1.66)	1.24	(0.93 – 1.64)	1.24	(0.93 – 1.64)	
Hyperglycemia							
Model 1	1.09	(0.83 – 1.42)	1.31	(1.01 – 1.71)	1.18	(0.90 – 1.54)	1
Model 2	1.08	(0.83 – 1.42)	1.31	(1.00 – 1.70)	1.18	(0.90 – 1.54)	
Model 3	1.05	(0.80 – 1.38)	1.28	(0.98 – 1.67)	1.15	(0.88 – 1.51)	
Model 4	1.14	(0.86 – 1.51)	1.33	(1.02 – 1.75)	1.17	(0.89 – 1.53)	
Hypertriglycemia							
Model 1	2.14	(1.61 – 2.85)	1.42	(1.06 – 1.91)	1.43	(1.06 – 1.91)	1
Model 2	2.16	(1.62 – 2.88)	1.44	(1.07 – 1.44)	1.43	(1.06 – 1.92)	
Model 3	2.12	(1.59 – 2.84)	1.44	(1.07 – 1.93)	1.43	(1.06 – 1.93)	
Model 4	2.14	(1.59 – 2.89)	1.44	(1.07 – 1.95)	1.43	(1.07 – 1.93)	
Low HDL							
Model 1	1.35	(1.05 – 1.73)	1.13	(0.88 – 1.46)	1.05	(0.82 – 1.35)	1
Model 2	1.36	(1.05 – 1.75)	1.15	(0.90 – 1.49)	1.05	(0.82 – 1.35)	
Model 3	1.38	(1.07 – 1.79)	1.17	(0.90 – 1.50)	1.06	(0.82 – 1.37)	
Model 4	1.39	(1.07 – 1.81)	1.17	(0.91 – 1.51)	1.06	(0.82 – 1.37)	

Model 1: age adjusted, Model 2: Model 1 + smoking, alcohol, exercise adjusted, Model 3: Model 2 + region, seasonality adjusted, Model 4: Model 3 + parathyroid hormone adjusted, *OR* odds ratio, *CI* confidence interval, *HDL* high density lipoprotein

## Discussion

The relationship between vitamin D levels and MetS has been widely studied in several populations [8, 11, 17, 18]. However, most studies have not exclusively examined this association in elderly participants. With aging, cutaneous vitamin D production is reduced [4]. With aging, there are reductions in the renal production of 1,25(OH)_2_D by the kidneys, calcium absorption, and the action of vitamin D receptors [19]. Moreover, MetS is a concern throughout the aged society [20], as it is related to insulin resistance and obesity. Further, Vitamin D is lipid-soluble and is sequestered in fat tissue, which leads to lower vitamin D levels in obesity [21]. Thus, a specific relationship is expected between vitamin D levels and MetS prevalence in the elderly.

In this community-based cohort study involving Korean men and women aged ≥65 years, we demonstrated that the risk of MetS was significantly higher in the lowest quartile compared to the highest quartile of the vitamin D level in both men and women. In particular, we found that a lower vitamin D level is significantly associated with a higher prevalence of high waist circumference, elevated triglyceride level, and low HDL level in both sexes independent of age, smoking status, alcohol drinking status, exercise status, region of residence, and seasonality.

Vitezova et al. revealed that higher 25(OH)D concentrations in the elderly were associated with lower prevalence of MetS and with higher HDL-cholesterol, lower triglyceride, lower waist circumference, and lower serum glucose [11]. However, in that study the results were combined for both men and women, and included both middle-aged and elderly adults; in contrast, in the present study, the results were stratified by sex, and the age of the study participants was ≥65 years. Vitezova et al. [11] also reported a significant effect modification by sex (P interactions < 0.05) in a subgroup analysis. They showed that a lower prevalence of high triglyceride level was significantly associated with high vitamin D level, similar to our findings.

In our study, MetS and its components, including hypertriglyceridemia and low-HDL level, were associated with vitamin D level in the lowest quartile group in both men and women in all models. However, the significance of the association between high waist circumference and vitamin D level in the lowest quartile group was maintained in men, but not in women. After additional adjustment by PTH level, the association between high waist circumference and vitamin D level was not maintained in men. Vitamin D deficiency causes elevation of PTH, which also affects the storage of fat [22]. Waist circumference is measured to reflect abdominal obesity; however, it does not completely represent the amount of visceral fat, which is more related to insulin resistance, and fat distribution, which could differ according to sex. Kim et al. [23] suggested sex-specific association of PTH and vitamin D with MetS. However, we did not observe a sex-specific effect on the association between vitamin D level and MetS, or between vitamin D level and specific MetS components. Bea et al. observed significant inverse relationships between 1,25(OH)_2_D and high triglyceride level; however, for 25(OH)D concentrations, significant inverse relationships were observed with MetS, waist circumference, and triglyceride [24]. In our sensitivity analysis for total population, 25(OH)D level as a continuous variable showed significant inverse relationships with MetS (OR 0.97, 95% confidence interval 0.96–0.98) by adjusting age, sex, smoking, alcohol, exercise, region of residence, seasonality, PTH. Oosterwerff et al. showed an increased risk of MetS in participants who were > 65 years of age with vitamin D level < 50 nmol in the Netherlands [25]. They showed that low HDL cholesterol levels and high waist circumference were related to vitamin D deficiency. In that study, both sexes were analyzed together. When we analyzed both sexes together, the risk of MetS and its components including high waist circumference, hypertriglyceridemia and low HDL were significantly higher in the lowest quartile compared to the highest quartile of the vitamin D level. An additional file 1 shows this in more detail (see Additional file 1). Compared with the other studies, a possible explanation for the distinct findings of our results may comprise differences in the ages of participants in the studies. The differences in results regarding the associations between vitamin D level and MetS components in young and middle-aged populations stratified on the basis of sex may be influenced by hormonal levels [10, 26]. In our study, no significant associations were observed between vitamin D levels and hypertension or hyperglycemia, which may have occurred because the participants included individuals already taking antihypertensive or antidiabetic drugs.

This study has certain limitations. First, this was a cross-sectional study. A meta-analysis has shown that while the correlation between serum vitamin D status and MetS prevalence in the general adult population has been established in cross-sectional studies, a significant relationship has not been reproduced in longitudinal studies [8]. However, in that meta-analysis the study populations of the included studies were heterogenous. The present cohort is scheduled to be followed-up. Thus, the longitudinal data obtained will be analyzed to explore the association between the change in vitamin D status and incidence of MetS in this population. Second, we could not consider factors such as calcium and vitamin D intake, which could have affected the serum levels of vitamin D. Nonetheless, the present study also has important strengths. Most previous studies included adults with age > 20 years or women in their 50s during the early-postmenopausal period. However, our study exclusively focused on the elderly Korean participants aged ≥65 years and our analyses were also stratified by sex. Thus, the effects of the vitamin D level were examined with little or no hormonal effect.

## Conclusions

In conclusion, vitamin D deficiency is associated with a higher prevalence of MetS in Korean elderly men and women. Adequate vitamin D level is important to decrease the risk of MetS. Further studies should evaluate the effect of vitamin D supplements on MetS prevalence in the elderly.

## Additional file

Additional file 1: Multivariable-adjusted odds ratio of metabolic syndrome and its components according to serum 25(OH)D levels stratified by body mass index (BMI). (DOCX 22 kb)

## Abbreviations

25(OH)D: 25-hydroxyvitamin D
HDL: High-density lipoprotein
HOMA-IR: Homeostatic model assessment-insulin resistance
MetS: Metabolic syndrom
NCEP-ATP III: National Cholesterol Education Program Adult Treatment Panel III
PTH: Parathyroid hormone
