# Correction to: Associations of serum 25-hydroxyvitamin D with metabolic syndrome and its components in elderly men and women: the Korean Urban Rural Elderly cohort study

**DOI:** 10.1186/s12877-019-1150-y

**Published:** 2019-05-08

**Authors:** Su Jin Lee, Eun Young Lee, Jung Hyun Lee, Jong Eun Kim, Kwang Joon Kim, Yumie Rhee, Hyeon Chang Kim, Yoosik Youm, Chang Oh. Kim

**Affiliations:** 1 Department of Internal Medicine, Severance Hospital, Endocrine Research Institute, Yonsei University College of Medicine, Seoul, South Korea; 2 Department of Preventive Medicine, Yonsei University College of Medicine, 50-1 Yonsei-ro, Seodaemun-gu, Seoul, South Korea; 3 Division of Endocrinology and Metabolism, Department of Internal Medicine, Seoul St. Mary’s Hospital, College of Medicine, The Catholic University of Korea, Seoul, South Korea; 4 Graduate School of Public Health Yonsei University, Seoul, South Korea; 5 Department of Sociology, Yonsei University, 50 Yonsei-ro, Seodaemun-gu, Seoul, South Korea; 6 Division of Geriatrics, Department of Internal Medicine, Yonsei University College of Medicine, 50-1 Yonsei-ro, Seodaemun-gu, Seoul, 03772 Republic of Korea

**Correction to: BMC Geriatr (2019) 19:102**

**https://doi.org/10.1186/s12877-019-1118-y**

Following the publication of this article [1], the authors reported an error in one of the author’s names. In this Correction the incorrect and correct author name are shown. The original publication of this article has been corrected.

Originally the author name was published as:

- Kwang Jun Kim

The correct author name is:

- Kwang Joon Kim
