# Supplementary material for: Associations of serum 25-hydroxyvitamin D with metabolic syndrome and its components in elderly men and women: the Korean Urban Rural Elderly cohort study
Source: BMC Geriatr. 2019 Apr 11;19:102. doi: 10.1186/s12877-019-1118-y (PMC6458686; doi:10.1186/s12877-019-1118-y)
Supplement: Supplementary file 1 — Multivariable-adjusted odds ratio of metabolic syndrome and its components according to serum 25(OH)D levels stratified by body mass index (BMI). (DOCX 22 kb) [file 12877_2019_1118_MOESM1_ESM.docx]

Supplement 1. Multivariable-adjusted odds ratio of metabolic syndrome and its components according to serum 25(OH)D levels stratified by body mass index (BMI)

|  | | | | | | | | | | | | | |
| --- | --- | --- | --- | --- | --- | --- | --- | --- | --- | --- | --- | --- | --- |
|  | | **Men** | | | | | | **Women** | | | | | |
| **Outcome** | **Vitamin D** | **BMI < 25** | | | **BMI ≥ 25** | | | **BMI < 25** | | | **BMI ≥ 25** | | |
|  |  | OR | Lower CI | Upper CI | OR | Lower CI | Upper CI | OR | Lower CI | Upper CI | OR | Lower CI | Upper CI |
|  |  |  | | |  |  |  |  |  |  |  |  |  |
| Metabolic syndrome | Q4 | 1 (reference) | | | 1 (reference) | | | 1 (reference) | | | 1 (reference) | | |
|  | Q3 | 1.571 | 0.862 | 2.863 | 1.195 | 0.62 | 2.302 | 1.205 | 0.866 | 1.676 | 1.661 | 1.051 | 2.624 |
|  | Q2 | 2.056 | 1.134 | 3.728 | 1.615 | 0.797 | 3.271 | 0.938 | 0.665 | 1.324 | 2.08 | 1.32 | 3.277 |
|  | Q1 | 2.33 | 1.242 | 4.368 | 1.803 | 0.891 | 3.645 | 1.511 | 1.069 | 2.135 | 1.552 | 0.983 | 2.449 |
|  |  |  |  |  |  |  |  |  |  |  |  |  |  |
| Abdominal Obesity | Q4 | 1 (reference) | | | 1 (reference) | | | 1 (reference) | | | 1 (reference) | | |
|  | Q3 | 2.488 | 1.134 | 5.46 | 1.247 | 0.611 | 2.546 | 1.253 | 0.908 | 1.73 | 0.979 | 0.439 | 2.185 |
|  | Q2 | 2.636 | 1.203 | 5.776 | 2.217 | 0.982 | 5.002 | 0.855 | 0.613 | 1.194 | 1.55 | 0.664 | 3.618 |
|  | Q1 | 2.038 | 0.876 | 4.739 | 1.399 | 0.652 | 3.002 | 0.901 | 0.64 | 1.269 | 1.075 | 0.468 | 2.468 |
|  |  |  |  |  |  |  |  |  |  |  |  |  |  |
| Hypertension | Q4 | 1 (reference) | | | 1 (reference) | | | 1 (reference) | | | 1 (reference) | | |
|  | Q3 | 0.894 | 0.549 | 1.457 | 1.382 | 0.601 | 3.18 | 1.135 | 0.805 | 1.6 | 1.287 | 0.759 | 2.18 |
|  | Q2 | 1.248 | 0.746 | 2.087 | 0.746 | 0.326 | 1.705 | 0.892 | 0.631 | 1.261 | 1.815 | 1.056 | 3.119 |
|  | Q1 | 0.722 | 0.426 | 1.226 | 1.936 | 0.792 | 4.731 | 1.175 | 0.815 | 1.694 | 1.11 | 0.654 | 1.882 |
|  |  |  |  |  |  |  |  |  |  |  |  |  |  |
| Hyperglycemia | Q4 | 1 (reference) | | | 1 (reference) | | | 1 (reference) | | | 1 (reference) | | |
|  | Q3 | 1.157 | 0.714 | 1.875 | 0.995 | 0.523 | 1.896 | 0.998 | 0.702 | 1.421 | 1.284 | 0.821 | 2.009 |
|  | Q2 | 1.29 | 0.792 | 2.101 | 2.029 | 1.014 | 4.062 | 1.123 | 0.786 | 1.605 | 1.437 | 0.926 | 2.23 |
|  | Q1 | 1.186 | 0.704 | 1.997 | 1.42 | 0.716 | 2.814 | 1.252 | 0.869 | 1.805 | 0.907 | 0.575 | 1.429 |
|  |  |  |  |  |  |  |  |  |  |  |  |  |  |
| Hypertriglycemia | Q4 | 1 (reference) | | | 1 (reference) | | | 1 (reference) | | | 1 (reference) | | |
|  | Q3 | 2.433 | 1.339 | 4.423 | 1.302 | 0.648 | 2.617 | 1.439 | 0.99 | 2.093 | 1.585 | 0.939 | 2.676 |
|  | Q2 | 2.824 | 1.536 | 5.19 | 1.209 | 0.575 | 2.539 | 1.125 | 0.758 | 1.671 | 2.041 | 1.232 | 3.383 |
|  | Q1 | 3.589 | 1.866 | 6.901 | 1.71 | 0.821 | 3.559 | 1.828 | 1.243 | 2.688 | 2.765 | 1.661 | 4.604 |
|  |  |  |  |  |  |  |  |  |  |  |  |  |  |
| Low HDL | Q4 | 1 (reference) | | | 1 (reference) | | | 1 (reference) | | | 1 (reference) | | |
|  | Q3 | 1.133 | 0.614 | 2.089 | 0.744 | 0.38 | 1.454 | 0.909 | 0.657 | 1.256 | 1.203 | 0.774 | 1.87 |
|  | Q2 | 1.666 | 0.921 | 3.013 | 0.849 | 0.418 | 1.727 | 1.115 | 0.801 | 1.551 | 1.113 | 0.723 | 1.713 |
|  | Q1 | 2.193 | 1.184 | 4.062 | 0.937 | 0.463 | 1.896 | 1.595 | 1.134 | 2.244 | 1.067 | 0.687 | 1.658 |
| * Adjusted by age, smoking, alcohol, exercise, region, seasonality, parathyroid hormone  Q1: quartile 1, Q2: quartile 2, Q3: quartile 3, Q4: quartile 4 | | | | | | | | | | | | | |
